# Interrelationship of childhood abuse, BIS/BAS, and stressful life events in predicting depression: A structural equation modeling study

**DOI:** 10.1002/pcn5.70210

**Published:** 2025-10-01

**Authors:** Yu Tamada, Osamu Takashio, Jiro Masuya, Masayuki Kikkawa, Rintaro Nibuya, Shunichiro Ito, Naoki Hashimoto, Hajime Tanabe, Takeshi Inoue

**Affiliations:** ^1^ Department of Psychiatry Tokyo Medical University Hachioji Medical Center Tokyo Japan; ^2^ Department of Psychiatry Tokyo Medical University Tokyo Japan; ^3^ Department of Psychiatry Hokkaido University Graduate School of Medicine Sapporo Japan; ^4^ Faculty of Humanities and Social Sciences Shizuoka University Shizuoka Japan

**Keywords:** childhood abuse, covariance structure analysis, depression, neglect

## Abstract

**Aim:**

Childhood abuse, personality traits, and stressful life events are among the many factors that contribute to the development of depression, yet the nature of their interrelationships has not been fully clarified. This research set out to investigate the interrelationships among childhood abuse, stressful life events, behavioral inhibition system (BIS)/behavioral activation system (BAS) sensitivity, and depressive symptoms in adults from a nonclinical population using structural equation modeling.

**Methods:**

A total of 286 Japanese adults without any psychiatric history participated in the study. Participants filled out a set of self‐report instruments, including the Patient Health Questionnaire‐9 (PHQ‐9), the BIS/BAS scale, the Child Abuse and Trauma Scale, and the Life Experiences Survey (LES). Data analysis was conducted using multiple regression and structural equation modeling.

**Results:**

By structural equation modeling, childhood abuse showed a significant indirect effect on PHQ‐9 through BIS. The pathway from childhood abuse to the LES negative change score via BIS reached statistical significance, while the remaining indirect routes did not show significance. Childhood abuse had a significant total indirect effect on PHQ‐9 scores (indirect coefficient = 0.092, *p* = 0.001).

**Conclusion:**

Childhood abuse influences the increase in depressive symptoms via increased BIS sensitivity, but not via stressful life events.

## Graphical abstract

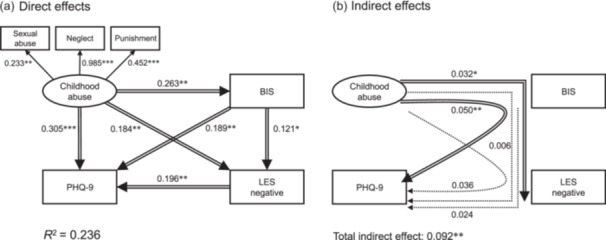

## INTRODUCTION

The development of depression is known to involve a variety of psychosocial factors. In particular, adverse childhood experiences, such as emotional neglect, and stressful life events in adulthood have consistently been reported as significant risk factors for depressive symptoms.^1^, ^2^, ^3^, ^4^, ^5^, ^6^, ^7^ Several studies have demonstrated that childhood neglect and maltreatment are strongly associated with depression in adulthood,^8^, ^9^ and that stressful life events, both causally and noncausally, influence depression onset.^2^ However, these factors likely do not operate in isolation, but rather interact with individual personality characteristics, shaping vulnerability to depression across the lifespan.^3^, ^6^, ^10^

To understand the mechanisms underlying these associations, it is necessary to examine how these external risk factors influence internal psychological processes. Personality traits may serve as important moderators or mediators of stress sensitivity and emotional regulation. While broad trait models such as the Big Five have been extensively used to characterize personality dimensions,^11^ they are largely descriptive and not specifically designed to explain motivational or affective responses to environmental stimuli. By contrast, within Gray's reinforcement sensitivity theory,^12^, ^13^, ^14^, ^15^ the behavioral inhibition system (BIS) and behavioral activation system (BAS) are understood to reflect individual differences in sensitivity to punishment and reward, respectively. These systems are believed to underlie tendencies toward negative and positive affect, and have been empirically linked to the development and course of depression.^16^, ^17^, ^18^, ^19^, ^20^, ^21^, ^22^, ^23^, ^24^, ^25^, ^26^, ^27^ Given that childhood abuse and stressful life events are experiences involving threat or loss of reward, BIS/BAS may serve as particularly relevant personality‐based mechanisms that mediate their psychological impact.

The BIS is a motivational system activated by cues associated with punishment, frustration, or the absence of reward, as well as by novel stimuli. It functions to inhibit behaviors that may lead to painful or negative outcomes. Therefore, BIS activation results in the suppression of goal‐directed movements. This mechanism is believed to play a role in triggering negative emotions, including sadness, anxiety, and fear, in reaction to these signals.^28^, ^29^

Conversely, the BAS is activated by cues signaling reward or the absence of punishment. The activity of this system facilitates goal‐oriented behavior. BAS is responsible for positive emotional experiences, such as hope, elation, and happiness. Higher BAS sensitivity is associated with greater effort toward achieving goals, and a tendency to experience positive emotions when encountering signals indicating that reward is imminent.^28^, ^29^ Individual differences in BIS and BAS sensitivity are considered temporally stable and are believed to have a strong genetic basis.^30^ However, environmental factors, such as adverse experiences in childhood, are also known to influence these systems.^31^, ^32^

A substantial body of evidence supports the association between BIS/BAS levels and specific psychiatric disorders.^16^, ^17^, ^18^, ^19^, ^20^, ^21^ High BIS levels^16^, ^20^, ^22^, ^23^, ^24^, ^25^ or low BAS levels^20^, ^23^, ^24^, ^25^, ^26^ are consistently associated with depression. Furthermore, lower BAS levels have been linked to an unfavorable prognosis in individuals with depression and are found to predict long‐term disease course.^23^, ^27^

Several studies have investigated the relationship between BIS/BAS levels, experiences of childhood abuse, and depression. Rosenman and Rodgers conducted a study on a general adult population and found that childhood domestic adversity increases BIS levels but has no significant association with BAS.^33^ In contrast, Miu et al. indicated that interpersonal trauma in childhood can contribute to increased BIS and decreased BAS, leading to increased depressive symptoms in adults.^34^ Huh et al. investigated the interaction between childhood trauma, BIS/BAS levels, and adult attachment styles in depressed individuals, and found that emotional neglect in childhood and BIS/BAS interactions contribute to attachment anxiety. Specifically, individuals with high BAS sensitivity who experienced emotional neglect in childhood demonstrated particularly high attachment anxiety.^35^ These studies suggest that childhood trauma increases BIS sensitivity, making individuals more prone to anxiety and depressive symptoms. However, regarding BAS, whereas biological theories propose that early adversity might blunt reward sensitivity,^36^ findings on the association between childhood trauma and BAS are inconsistent across studies.

As far as we are aware, no prior research has examined the interrelationships between childhood abuse, BIS/BAS sensitivity, stressful life events, and depressive symptoms. We hypothesized that childhood abuse impacts depressive symptoms through changes in BIS/BAS levels and the mediation of stressful life events. This study aimed to examine whether BIS/BAS levels and stressful life events mediate the relationship between depressive symptoms and childhood abuse in adult volunteers, using structural equation modeling.

## METHODS

### Subjects

A total of 294 Japanese adult volunteers were recruited from the community between July 2011 and December 2011 using a convenience sampling method. Participants were approached through flyers, word of mouth, and acquaintances of the researchers at Hokkaido University Hospital in the Sapporo area. Individuals with a self‐reported history of psychiatric disorders were excluded from the study. Written informed consent was obtained from all participants before they took part in the study. Following the exclusion of eight participants because of significant missing data, 286 individuals were enrolled in the study. We determined the target sample size based on conventional guidelines for structural equation modeling. Considering that our model included 21 free parameters and applying a conservative rule of at least 12 cases per parameter, the required sample size was estimated to be at least 252, consistent with previous recommendations that at least 200 participants are generally required for stable SEM estimation.^37^ Our final sample of 286 participants exceeded this target. The questionnaires were distributed to participants in person by the research staff. The survey included four questionnaires, as outlined below, together with a demographic survey that collected information on age, sex, education, employment status, marital status, family structure, family history of psychiatric disorders, and comorbid physical illness. To ensure complete confidentiality, participants returned the questionnaires anonymously by mail to the research team. Ethical approval for this study was obtained from the Hokkaido University Hospital Institutional Review Board (approval No. 010‐0041), and all procedures complied with the principles of the Declaration of Helsinki.

### Measures

#### Patient Health Questionnaire‐9

The Patient Health Questionnaire‐9 (PHQ‐9), a self‐report tool developed by Spitzer et al.,^38^ is designed for screening depressive symptoms. It includes nine items that align with the diagnostic criteria for MDD in the DSM‐IV.^39^ This scale also serves as a measure of depression severity.^40^ The Japanese version of the PHQ‐9^41^ has shown strong internal consistency (Cronbach's *α* = 0.93) and satisfactory construct validity.^42^

#### BIS/BAS scale

The BIS/BAS scale created by Carver and White^28^ was employed to assess BIS/BAS sensitivity. Containing 20 items, the instrument is completed by participants themselves and employs a 4‐point Likert scale, with options from “strongly disagree” to “strongly agree.” The BIS component includes seven items indicating tendencies to avoid punishment, whereas the BAS component comprises three subscales, namely fun‐seeking, drive, and reward responsiveness, with a total of 13 items. The Japanese version of the BIS/BAS scale showed acceptable internal consistency in a previous study (Cronbach's *α* = 0.80 for BIS and 0.81 for BAS total) and replicated the original four‐factor structure.^43^

#### Child Abuse and Trauma Scale

The Child Abuse and Trauma Scale (CATS) is a self‐report tool comprising 38 items, designed to retrospectively evaluate experiences of psychological maltreatment during childhood and adolescence.^44^ The frequency of particular abuse experiences in childhood and adolescence is rated by participants on a 0 (never) to 4 (always) scale. The scale includes the following three subscales: sexual abuse, negative home environment/neglect, and punishment, with each subscale score calculated as the average of the items it comprises. The reliability of the Japanese version of the CATS^45^, ^46^ is supported by a Cronbach's *α* of 0.91 for the total score, along with evidence of satisfactory factorial validity.

#### Life Experiences Survey

The Life Experiences Survey (LES) is a 57‐item self‐report tool designed to evaluate participants' experiences of life events that caused notable changes in their lives over the previous year.^47^ Participants assess the impact of every event using a 7‐point scale, where scores range from −3 (extremely negative) to 3 (extremely positive). The Japanese version of the LES^10^ was utilized in this study. In the Japanese version of the LES, test–retest reliability was supported by moderate intraclass correlation coefficients of 0.47 (positive change score) and 0.45 (negative change score). In addition, good construct validity was observed.^10^

### Data analysis

First, Spearman's rank correlation coefficient analysis and the Mann–Whitney *U*‐test were used to examine the relationships and associations between psychological questionnaire and demographic data and the PHQ‐9 score. Second, using the PHQ‐9 total score as the dependent variable, a forced‐entry multiple regression analysis was conducted, incorporating as independent variables those significantly associated with the PHQ‐9 from the previous analysis. Missing values occurred only in one binary variable (“living alone”), with a missing rate of 3.8%. According to Little's test for missing completely at random (MCAR), the data were determined to be MCAR. Therefore, we applied listwise deletion in the multiple regression analysis. To assess multicollinearity, the variance inflation factor (VIF) was calculated for all predictors. A VIF value above 10 was considered indicative of multicollinearity. Third, to support the construction of the structural equation model (SEM), we examined Pearson's correlations among five key observed variables: the neglect, punishment, and sexual abuse subscales of the CATS, the BIS score, and the negative change score of the LES (LES‐N score). Type I error related to multiple comparisons was controlled using the Bonferroni correction; for the ten correlation tests, statistical significance was defined as *p* < 0.005. Finally, the SEM was constructed based on the findings from the prior correlation and multiple regression analyses. A non‐parametric bootstrapping method with 5000 resamples was used to evaluate the significance of indirect effects within the SEM. We computed bias‐corrected 95% confidence intervals (CIs), considering indirect effects statistically significant if the 95% CI excluded zero. Model fit was evaluated using several indices, including the root mean square error of approximation (RMSEA), the comparative fit index (CFI), and the Tucker–Lewis index (TLI). Following standard criteria, a good model fit was defined as an RMSEA less than 0.05, a TLI exceeding 0.97, and a CFI exceeding 0.97.^48^ For the SEM analysis, the coefficients were standardized to fall within a range of −1 to 1.

spss Statistics version 28 software was used to perform Spearman's rank correlation coefficient analysis, the Mann–Whitney *U*‐test, and multiple regression analysis. Mplus 8.5 software was used to perform the SEM. A statistically significant difference between groups was defined as a *p*‐value of less than 0.05.

## RESULTS

The PHQ‐9 total score was significantly higher among participants who were female, lived alone, or were unmarried. PHQ‐9 total scores showed significant correlations with both the neglect and total scores on the CATS, as well as with the BIS score and the LES‐N scores (Table 1).

**Table 1:** Characteristics, and Patient Health Questionnaire‐9 (PHQ‐9), Child Abuse and Trauma Scale (CATS), behavioral inhibition system (BIS)/behavioral activation system (BAS), and Life Experiences Survey (LES) scores and their correlation with or effects on PHQ‐9 scores.

Characteristic or measure		Value (number or mean ± SD)	Correlation with PHQ‐9 (*ρ*) or effect on PHQ‐9 (mean ± SD of PHQ‐9 scores, *U*‐test)
Age, years		42.4 ± 11.3	*ρ* = − 0.12, n.s.
Sex (male:female)		168:118	Male (2.7 ± 3.2) versus female (4.4 ± 4.1)**
Education, years		14.9 ± 2.2	*ρ* = − 0.11, n.s.
Employment status (employed:unemployed) observed		236:47	Employed (3.3 ± 3.7) versus unemployed (3.4 ± 3.5)
Homemakers of unemployed persons		41	n.s.
Marital status	Married	223	Married (2.9 ± 3.4) versus unmarried (5.0 ± 4.2)**
	Unmarried	63	
Living alone (yes:no)		61:214	Yes (3.9 ± 3.1) versus no (3.2 ± 3.8)*
Number of offspring		1.4 ± 1.0	*ρ* = − 0.11, n.s.
Presence of offspring (yes:no)		203:83	Yes (3.2 ± 3.7) versus no (4.0 ± 3.7), n.s.
Comorbidity of physical disease (yes:no) medical disease		63:223	Yes (3.3 ± 3.6) versus no (3.4 ± 3.7), n.s.
First‐degree relative with psychiatric disorders (yes:no)		43:243	Yes (3.7 ± 3.8) versus no (3.3 ± 3.7), n.s.
PHQ‐9 total score		3.4 ± 3.7	
CATS (average score)	Neglect	0.56 ± 0.65	*ρ* = 0.31**
	Punishment	1.49 ± 0.64	*ρ* = 0.06, n.s.
	Sexual abuse	0.03 ± 0.10	*ρ* = 0.02, n.s.
	Total	0.65 ± 0.46	*ρ* = 0.25**
BIS/BAS (average score)	BIS	18.21 ± 4.04	*ρ* = 0.29**
	BAS‐D	10.56 ± 2.87	*ρ* = 0.01, n.s.
	BAS‐FS	9.45 ± 2.74	*ρ* = 0.09, n.s.
	BAS‐RR	14.55 ± 3.03	*ρ* = 0.02, n.s.
	BAS total	34.57 ± 7.76	*ρ* = 0.05, n.s.
LES (change score)	Negative	2.28 ± 4.31	*ρ* = 0.29**
	Positive	2.06 ± 3.42	*ρ* = 0.08, n.s.

*Note*: Data are presented as means ± SDs or numbers. *ρ* = Spearman's rank correlation coefficient.

Abbreviations: D, drive; FS, fun‐seeking; n.s., not significant; RR, reward responsiveness; SD, standard deviation.

* *p* < 0.05

** *p* < 0.01.

Based on the results from Spearman's rank correlation coefficient analysis and the Mann–Whitney *U*‐test in Table 1, variables significantly associated with the total PHQ‐9 score were subsequently analyzed using forced‐entry multiple regression analysis. The dependent variable was the total PHQ‐9 score, and the independent variables included marital status (unmarried = 1, married = 2), sex (male = 1, female = 2), living alone (no = 1, yes = 2), CATS neglect score, BIS score, and LES‐N score. Although the CATS total score was significantly correlated with PHQ‐9 score (Table 1), it was omitted from the multiple regression analysis owing to its strong correlation with the CATS neglect score (*ρ* = 0.83). The results are presented in Table 2. The BIS score, CATS neglect score, and LES‐N score were found to be significant predictors of the PHQ‐9 score (adjusted *R*² = 0.26, *F* = 17.3, *p* < 0.001). Multicollinearity was ruled out.

**Table 2:** Results of multiple regression analysis of Patient Health Questionnaire‐9 (PHQ‐9) scores.

Independent variable	Beta	*p* value	VIF
Neglect score of CATS	0.29	<0.001	1.22
Negative change score of LES	0.21	<0.001	1.07
BIS score	0.19	<0.001	1.13
Sex	0.05	0.35	1.17
Living alone	0.003	0.96	1.19
Marital status	–0.09	0.14	1.37

*Note*: Beta = standardized partial regression coefficient. Dependent variable: PHQ‐9 summary score. Six independent variables: sex (female = 2, male = 1), marital status (married = 2, unmarried = 1), living alone (yes = 2, no = 1), neglect score of CATS, BIS score, and negative change scores of LES. Adjusted *R* ^2^ = 0.26; *F* = 17.3, *p* < 0.001.

Abbreviations: BIS, behavioral inhibition system; CATS, Child Abuse and Trauma Scale; LES, Life Experiences Survey; VIF, variance inflation factor.

Table 3 presents the correlations among CATS subscale scores, BIS/BAS subscale scores, and LES subscale scores. Among the subscales of childhood abuse, neglect was positively correlated with BIS and all subscales of BAS, whereas punishment and sexual abuse did not show significant correlations with BIS/BAS. Neglect showed a positive correlation with the LES‐N score. BIS was positively correlated with both the positive and LES‐N score, whereas all BAS subscales exhibited positive correlations with the positive change score of LES.

**Table 3:** Correlation (*ρ*) between Child Abuse and Trauma Scale (CATS), behavioral inhibition system (BIS), and negative change score of Life Experiences Survey (LES).

	Neg	Pun	Sex	BIS	LES‐N
Neg	1.00	0.32*	0.21*	0.24*	0.20*
Pun		1.00	0.11	0.12	0.07
Sex			1.00	0.01	0.12
BIS				1.00	0.26*
LES‐N					1.00

*Note*: *ρ* = Spearman's rank correlation coefficient.

Abbreviations: BIS, behavioral inhibition system; LES‐N, negative change score of Life Experiences Survey; Neg, neglect subscale; Pun, punishment subscale; Sex, sexual abuse subscale.

* *p* < 0.005 (Bonferroni‐corrected).

**Figure 1:**
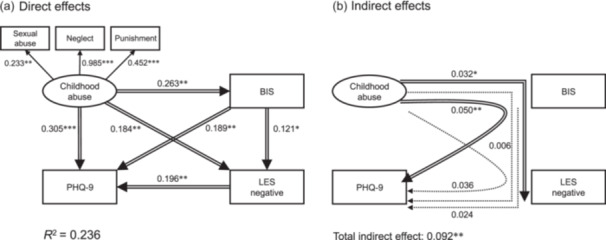
Results of covariance structure analysis in the structural equation model (SEM) with childhood abuse, behavioral inhibition system (BIS), stressful life events (Life Experiences Survey [LES]), and depressive symptoms (Patient Health Questionnaire‐9 [PHQ‐9]) scores. The results of direct (a) and indirect (b) effects of covariance structure analysis of 286 subjects from the nonclinical adult population with Child Abuse and Trauma Scale (CATS) scores, BIS score, negative change score of LES, and PHQ‐9 score. Rectangles indicate the observed variables, and the oval indicates the latent variable, which consists of three observed variables, namely, sexual abuse, neglect, and punishment. The arrows with double lines represent the statistically significant paths, and the arrows with broken lines represent the nonsignificant paths. The numbers beside the arrows show the standardized path coefficients (minimum –1, maximum +1). *p < 0.05; **p < 0.01; and ***p < 0.001.

To investigate the interrelationships among childhood abuse, stressful life events, BIS/BAS levels, and depressive symptoms, SEM analysis was conducted. The SEM was constructed with reference to the results of both the multiple regression analysis (Table 2) and the correlation analysis among the five observed variables included in the model (Table 3). Figure 1 presents the outcomes of the standardized path coefficients. In this model, BIS is situated between “adult life events” and “childhood abuse,” consistent with our hypothesis. We hypothesized BIS to function as a mediator between childhood abuse and depressive symptoms. A good model fit was indicated by the model fit indices (RMSEA = 0.000, TLI = 1.000, and CFI = 1.000). Since no modification indices above 10.000 were found, the model appeared to be adequately specified.

In the model presented in Figure 1a, the neglect subscale score had a notably high standardized coefficient in relation to the latent variable “childhood abuse.” Childhood abuse showed significant positive direct effects on BIS, the LES‐N score, and the PHQ‐9. Significant positive direct effects of BIS were observed on the LES‐N score and PHQ‐9 score, whereas the LES‐N score had a significant positive direct effect on the PHQ‐9 score.

The indirect effects are shown in Figure 1b. A significant indirect effect of childhood abuse on PHQ‐9 was observed through BIS. A significant indirect path from childhood abuse to the LES‐N score through BIS was found, while no other indirect paths were significant. A significant total indirect effect of childhood abuse on PHQ‐9 was observed (indirect path coefficient = 0.092, *p* = 0.001). Bootstrapping analysis (5000 resamples) confirmed that the indirect effect of childhood abuse on depressive symptoms via BIS was significant, as the 95% bias‐corrected CI did not include zero (95% CI [0.041, 0.172]).

## DISCUSSION

This study found that childhood abuse can lead to elevated depressive symptoms indirectly by increasing BIS sensitivity. Additionally, childhood abuse was found to influence the increase in depressive symptoms through heightened BIS sensitivity, but not via stressful life events. This study is, to our knowledge, the first to apply SEM in examining the interrelationships between childhood abuse, BIS/BAS, stressful life events, and depressive symptoms.

Previous research has indicated that interpersonal trauma in childhood impacts depressive symptoms through BIS activation,^34^ and our findings support this association. This may partly explain why childhood abuse has long‐term psychological effects. Although BIS/BAS levels can be influenced by environmental factors, they are also known to be genetically determined and relatively stable over time.^23^, ^30^ Childhood adversity is thought to heighten vulnerability, increasing sensitivity to psychological stress^7^; the BIS/BAS theory may represent a facet of this vulnerability. Additionally, a possible explanation for the role of BIS in exacerbating depressive symptoms is that individuals with high BIS sensitivity may rely on maladaptive cognitive‐emotional regulation strategies, such as rumination.^49^

As shown in Figure 1, childhood abuse significantly affects stressful life events via BIS; however, contrary to our hypothesis, the indirect path from BIS to depressive symptoms through stressful life events was not significant. The SEM in this study suggests that childhood abuse may heighten vulnerability to stressful life events through BIS. However, although BIS was modeled as a mediator, our results indicate that childhood abuse does not affect depressive symptoms through the sequential path involving both BIS and stressful life events.

Although earlier studies^20^, ^23^, ^24^, ^25^, ^26^ have reported a relationship between depression and reduced BAS levels, the present study did not find a significant association between BAS levels and depressive symptom severity. Low BAS levels have been suggested to be associated with a relatively severe subtype of anhedonic depression.^24^, ^50^ This lack of association in our study may be owing to the fact that our sample consisted of a nonclinical adult population, which did not include individuals with severe depression. In the study by Miu et al.,^34^ although their sample was also drawn from an adult population, childhood trauma was shown to potentially increase depressive symptoms by being mediated via a reduction in BAS sensitivity, which was different from our results. Miu et al. focused only on severe trauma, defined as a score of 6 or higher out of 7, which may have led to stronger emotional influences on decreasing BAS. Additionally, the characteristics of Miu et al.'s sample, namely, relatively young age (18–40 years) and predominantly female (88.3%), differ from those of our study, which may have also contributed to the discrepancy in the findings.

Our findings suggest that heightened BIS sensitivity may play a key role in the development of depressive symptoms. From a clinical perspective, assessing BIS sensitivity could help guide the selection or tailoring of treatment strategies. For example, individuals with elevated BIS may benefit more from CBT components that specifically target avoidance and threat sensitivity, such as cognitive restructuring and exposure‐based techniques. Future research is needed to determine whether BIS‐informed personalization of CBT enhances treatment outcomes.

This study has several limitations. First, the use of convenient sampling may have introduced selection bias, limiting the generalizability of the findings. Second, it was performed on a nonclinical adult population, which included a large number of healthy individuals, limiting the applicability of our model to clinical patients with depression. Third, childhood abuse was evaluated retrospectively based on participants' recollections, which could have introduced recall bias. Finally, since this study utilized cross‐sectional data, causal relationships cannot be determined.

To overcome these limitations, future studies should adopt longitudinal designs to elucidate the causal relationships among childhood abuse, BIS/BAS sensitivity, and depressive symptoms. Additionally, clinical studies comparing individuals with diagnosed depression to nondiagnosed populations would help elucidate whether the patterns observed in this study generalize to clinical settings. Finally, cross‐cultural investigations are warranted to determine whether these findings, derived from a Japanese sample, are applicable across different cultural contexts.

In conclusion, we found that childhood abuse indirectly increases depressive symptoms via heightened BIS sensitivity, but not via stressful life events.

## AUTHOR CONTRIBUTIONS

**Yu Tamada**: Conceptualization; validation; formal analysis; methodology; investigation; visualization; writing—original draft; writing—review and editing. **Osamu Takashio**: Conceptualization; formal analysis; validation; visualization; writing—review and editing. **Jiro Masuya**: Data curation; writing—original draft. **Masayuki Kikkawa**: Data curation; writing—original draft. **Rintaro Nibuya**: Data curation; writing—original draft. **Shunichiro Ito**: Data curation; writing—original draft. **Naoki Hashimoto**: Data curation; writing—original draft. **Hajime Tanabe**: Investigation; methodology. **Takeshi Inoue**: Conceptualization; data curation; validation; formal analysis; investigation; methodology; project administration; writing—original draft; writing—review and editing; supervision. All authors contributed to and have approved the final manuscript.

## CONFLICT OF INTEREST STATEMENT

Yu Tamada has received honoraria from Eisai, Meiji Seika Pharma, MSD, Otsuka Pharmaceutical, and Sumitomo Pharma. Osamu Takashio has obtained personal fees from Eisai, EA Pharma, Janssen Pharmaceutical, Kyowa Pharmaceutical Industry, Meiji Seika Pharma, MSD, Otsuka Pharmaceutical, Sumitomo Pharma, Takeda Pharmaceutical, and Viatris Pharmaceuticals Japan. Jiro Masuya has received personal remuneration from Astellas, Eli Lilly, Meiji Yasuda Mental Health Foundation, and Otsuka Pharmaceutical, as well as research funding from Pfizer. Naoki Hashimoto has obtained personal fees from Janssen Pharmaceutical K.K., Kyowa Pharmaceutical Industry, Meiji Seika Pharma, Nippon Boehringer Ingelheim Co., Otsuka Pharmaceutical, Sumitomo Pharma, Takeda Pharmaceutical, and Yoshitomiyakuhin, and has served as a consultant for Nippon Boehringer Ingelheim Co. Takeshi Inoue has received personal remuneration from Daiichi Sankyo, Eli Lilly, Janssen Pharmaceutical, Mochida Pharmaceutical, MSD, Taisho Toyama Pharmaceutical, Takeda Pharmaceutical, and Yoshitomiyakuhin; research grants from Astellas, Eisai, Shionogi, and Tsumura; and both research funding and personal fees from Kyowa Pharmaceutical Industry, Meiji Seika Pharma, Mitsubishi Tanabe Pharma, Novartis Pharma, Otsuka Pharmaceutical, Pfizer, and Sumitomo Pharma. He has also served on the advisory boards of Mitsubishi Tanabe Pharma, Novartis Pharma, and Pfizer. The other authors state that they have no commercial or financial relationships that could be interpreted as potential conflicts of interest in relation to this research.

## ETHICS APPROVAL STATEMENT

This study protocol was reviewed and approved by the institutional review board of Hokkaido University Hospital (study approval number: 010‐0041).

## PATIENT CONSENT STATEMENT

All individuals who took part in the study gave their written informed consent.

## CLINICAL TRIAL REGISTRATION

N/A.

## Data Availability

Data supporting the findings of this study can be obtained from the corresponding author upon reasonable request. Due to privacy and ethical considerations, these data are not publicly accessible.
